# CFD–DEM modelling of particle entrainment in wheel–rail interface: a parametric study on particle characteristics

**DOI:** 10.1007/s40534-024-00365-1

**Published:** 2025-01-15

**Authors:** Sadaf Maramizonouz, Sadegh Nadimi, William Skipper, Roger Lewis

**Affiliations:** 1 School of Engineering, Newcastle University, Newcastle upon Tyne, NE1 7RU UK; 2 Leonardo Centre for Tribology, Department of Mechanical Engineering, University of Sheffield, Sheffield, S1 3JD UK

**Keywords:** Rail-sanding, CFD–DEM coupling, Numerical analysis, Particle characteristics

## Abstract

To mitigate and alleviate low wheel–rail adhesion, a train-borne system is utilised to deposit sand particles into the wheel–rail interface via a jet of compressed air in a process called rail-sanding. Britain Rail Safety and Standards Board introduced guidelines on the sand particles’ shape, size, and uniformity which needs to be adhered to for rail-sanding. To further investigate these guidelines and help improve them, this research presents a parametric study on the particle characteristics that affect the rail-sanding process including density, size and size distribution, coefficient of uniformity, and shape, utilising a coupled computational fluid dynamics–discrete element method (CFD–DEM) model. The efficiency of rail-sanding is estimated for each case study and compared to the benchmark to optimise the sand characteristics for rail-sanding. It is concluded that particle size distribution (within the accepted range) has an insignificant effect on the efficiency while increasing particle size or the coefficient of uniformity decreases the efficiency. Particle shape is shown to highly affect the efficiency for flat, compact and elongated particles compared to the spherical shape. The current numerical model is capable of accurately predicting the trends in the efficiency compared to the actual values obtained from full-scale experiments.

## Introduction

The British railway industry annually spends around £345 million to mitigate the consequences of traction/adhesion^1^ loss between the train’s wheel and the rail. Low adhesion can occure due to the existence of a third body layer such as water, oil, and leaf on or bonded to the top of the rail. Adhesion loss during the train’s acceleration and deceleration phases can result in train delays, safety risks, and in the worst-cases train collisions [1, 2].

To improve the railway transportation by increasing the wheel–rail adhesion, a train-borne system is utilised to deposit sand particles into the wheel–rail interface via a jet of compressed air to control the behaviour of the granular material. This process is called rail-sanding and is reliable in alleviating the effects of adhesion loss [1–4]. However, in some circumstances, rail-sanding offers an efficiency of only < 20% which corresponds to > 80% of the deposited sand particles not being entrained into the wheel–rail interface thus playing no role in increasing the adhesion [5]. This means that the rail-sanding system can potentially be redesigned to optimise the efficiency of sand entrainment in the wheel–rail interface and thus reducing the risks of railway transportation as well as enhancing the sustainability of sand usage considering the limited resources of this natural material [1, 3, 6].

The rail-sanding process is often studied experimentally in controlled environments via twin-disc set-ups [3, 7, 8], and full-scale laboratory test rigs [5], or by performing field tests [9]. Although these tests are capable in offering accurate data, they can be expensive and inaccessible for the wider community. Another approach to study the rail-sanding process is using computational modelling which can provide accurate data in a less expensive and more accessible manner [10–12]. The dynamics of sand can be simulated using the discrete element method (DEM) [10, 11] which can then be coupled to computational fluid dynamics (CFD) in either a two-way [13] or a one-way [14] approach to model the fluid-particle interactions. In our previous studies, first, DEM simulations of rail-sanding for various sander positions was validated by the experimental data presented by Lewis et al. [5]; and second, a one-way coupled CFD–DEM model was employed for a parametric study of the train characteristics that affect the efficiency of sand application (entrainment efficiency) such as the train velocity, the sand flow rate, and the nozzle geometry [14].

As a continuation of the previous research [14], the current paper presents a parametric study on the particle characteristics that affect the rail-sanding process. For this purpose, a coupled CFD-DEM model is utilised to simulate the rail-sanding process, the entrainment efficiency of sand particle is estimated for each case study and compared to the benchmark case and other cases to find the optimum sand for rail-sanding. The finding of this study can be used to refine the guidelines defining the characteristics of the sand particles used for rail-sanding. These guidelines are set by the organisation responsible for the safety of railway transport in each country. Here, the guidelines introduced by Great Britain Rail Safety and Standards Board (RSSB) in the second issue of GMRT2461 document are outlined as follows [15]:

- The sand shall, as far as possible, consist of rounded irregular shaped grains.
- The maximum proportion of grains of diameter <0.71 mm (22 British standard sieve mesh) shall be not more than 5% by weight.
- The maximum proportion of grains of diameter >2.8 mm (5 British standard sieve mesh) shall be not more than 5% by weight.
- The coefficient of uniformity (CoU) shall be less than 1.5. The CoU is defined as the ratio of the sand diameter at which 60% of the sand weight is finer to the corresponding value at 10% finer.

## Methodology

### Discrete element method

The discrete element method is used to simulate the behaviour of granular materials. This method employs the simple, but crucial, assumption of the particles being rigid discrete objects. Newton’s and Euler’s laws of translational and rotational motions [16] are then used to model the mechanical behaviour of the particles with the governing equations appearing as follows:

1 $$m_{i} \frac{\text{d}v_{i}}{\text{d}{t}}\sum F_{\text{C}i} + m_{i} g + F_{\text{D}i},$$

2 $$\frac{\text{d}(I_{i}\cdot \omega_{i})}{\text{d}t} = \sum M_{{\text{C}_{i} }},$$

where ${m}_{i}$, ${I}_{i}$, ${v}_{i}$, and ${\omega }_{i}$ are the mass, the moment of inertia, the translational and the rotational velocities of the individual particle *i*, respectively; $t$ is time and $g$ is the gravitational acceleration; ${{F}_\text{C}}_{i}$ and ${M}_{\text{C}i}={R}_{i} \times {F}_{\text{C}i}$ are, respectively, the contact force and torque between each particle and its neighbouring particles and walls, ${R}_{i}$ is the particle’s radius [16], and ${{F}_\text{D}}_{i}$ is the drag force acting on the particle.

Particles interact with each other and the walls of the structures surrounding them. To model these interactions a suitable contact model should be used. In the present work, the elastic contact force, i.e. Hertz–Mindlin contact model, is chosen [16]. This contact model was shown to be accurate enough for the purposes of the current research [11].

### Computational fluid dynamics

The continuity and Navier–Stokes equations [17] are used to model the dynamical behaviour of the fluid medium including both the jet of air that deposits the sand particles and the relatively stationary air surrounding all. These equations are presented as follows:

3 $$\nabla \cdot \left( {{\varvec{U}}_{{\text{f}}} } \right) = 0,$$

4 $$\rho_{{\text{f}}} \left( {\frac{{\partial {\varvec{U}}_{{\text{f}}} }}{\partial t} + \left( {{\varvec{U}}_{{\text{f}}} \cdot \nabla } \right){\varvec{U}}_{{\text{f}}} } \right) = - \nabla P + \mu_{{\text{f}}} \left( {\nabla^{2} {\varvec{U}}_{{\text{f}}} } \right),$$

where ${{\varvec{U}}}_{\text{f}}$, ${\rho }_{\text{f}}$, and ${\mu }_{\text{f}}$ are the fluid’s velocity vector, density, and viscosity, respectively, and $P$ is the pressure [17].

### CFD-DEM coupling

The one-way coupling approach is chosen to link the DEM and CFD simulations together. This means that dynamics of the fluid affects the particle behaviour but not vice versa which is a reasonable assumption due to small sizes and low velocities of the particles compared to the whole system. This one-way coupled CFD-DEM modelling is performed by utilisation of the drag force acting on the sand particles, ${F}_{\text{D}}$:

5 $$F_{\text{D}i} = \frac{1}{2}\rho_\text{f} C_\text{D} A\left| {v_{i} - U_\text{f} } \right|\left( {v_{i} - U_\text{f} } \right),$$

where ${C}_{\text{D}}$ is the particles drag coefficient which is a function of the particle’s Reynolds number ${(Re)}$ and shape, and $A$ is the reference area of the particle. In most cases, the drag force dominates the majority of the particle dynamics [17, 18].

It was shown in our previous research that among the various models already presented in the literature for estimating the drag force, the model proposed by Ganser is versatile and results in a desirable accuracy in estimation of the drag force for various particle shapes [19]. So, the Ganser drag model [20] is utilised to estimate the particle’s drag coefficient in the current research. The Ganser drag model uses the true sphericity of the particle, ${\phi }_{\text{W}}={A}_{\text{sph}}/{A}_{\text{p}}$, defined by Wadell [21] as the ratio of surface area of the volume equivalent sphere (${A}_{\text{sph}}$) to the particle surface area (${A}_{\text{p}}$) to evaluate the drag coefficient of each particle:

6 $$C_\text{D}= \frac{24K\text{s}}
{{Re}}\left(1+0.1118\left(\frac{{Re}K_\text{N}}{K \text{s}}\right)^{0.6567}\right)+ \left(\frac{0.4305K_{\text{N}}}{1+\frac{3305}{\frac{{Re}K_\text{N}}{K\text{S}}}}\right),$$

7 $${{Re}} = \rho_{{\text{f}}} \left| {{\varvec{v}}_{i} - {\varvec{U}}_{{\text{f}}} } \right|\frac{d}{\mu}_{{\text{f}}},$$

8 $$K_{{\text{N}}} = 10^{{1.8148\left( { - {\text{log}}\phi_{{\text{W}}} } \right)^{0.5743} }},$$

9 $$K_{{\text{S}}} = \frac{1}{3} + \left( {\frac{2}{3}\sqrt {\phi_{{\text{W}}} } } \right),$$

10 $$\phi_{{\text{W}}} = \frac{A_\text{sph}}{A_\text{p}},$$

where ${K}_{\text{S}}$ and ${K}_{\text{N}}$ are Stoke’s and Newtons’s drag correction factors, respectively, and $d$ is the reference length of the particle.

The one-way CFD-DEM simulation is performed by first exporting the steady-state velocity field of the fluid from the CFD simulations and then importing it into the DEM model. After that, the chosen drag model is implemented in the DEM simulation to calculate the drag force on each discrete particle at each time step using the fluid velocity field and the dynamic and geometric properties of each particle.

### Modelling the particle shape

The shape of natural sand grains used in rail-sanding application is rarely spherical. In classic DEM models, the shape of the particles is often simplified to spheres due to the already established contact detection algorithms and contact models for spherical particles. To represent particles’ irregular shapes in DEM models, and still be able to utilise the established contact detection algorithms and contact models, the complex geometries of the particles can be represented as clumps made of a number of either overlapping or non-overlapping spherical elements. This modelling approach can also retain the fine details of the particle’s shape including local roundness, roughness, and concavities [22].

**Fig. 1:**
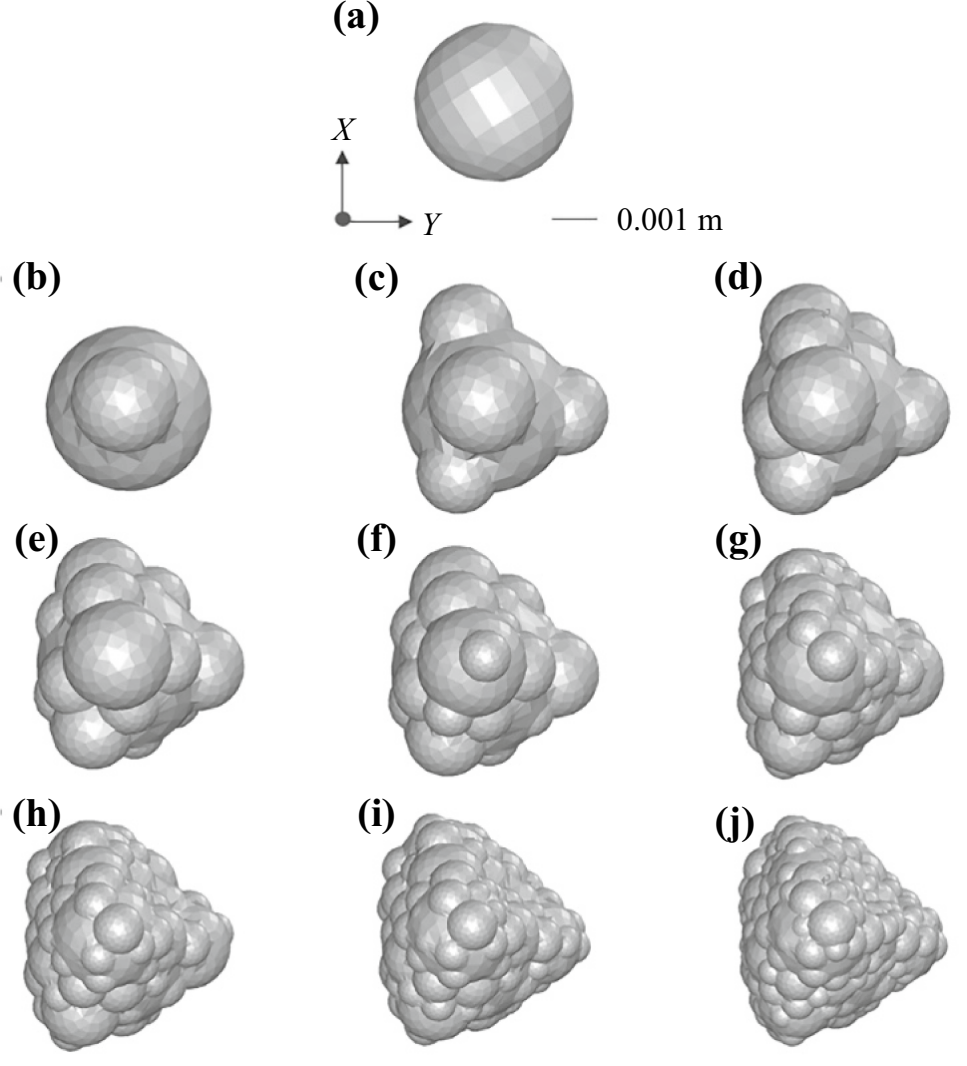
Compact sand particle shape represented as a clump of **a–j** 1, 2, 5, 10, 20, 30, 50, 70, 100, and 200 spherical elements at the orientation resulting in the maximum value of the drag coefficient for the actual shape

The number of the spherical elements used to represent the particle shape can significantly affect the accuracy and the cost of the computational simulation. To find the optimum number of spherical elements for the present study to accurately represent the particle shape without dramatically increasing the computational cost, irregular particles of different shapes are represented as multi-spheres clumps of ten different element numbers (one, two, five, ten, twenty, thirty, fifty, seventy, one-hundred, and two-hundred) generated by the Euclidean method utilising the CLUMP code [22]. The irregular particle shapes are classified in four shape categories of compact, flat, elongated, and bladed [23] based on the framework introduced by Zingg [24] and refined by Angelidakis et al. [23]. The multi-sphere representations of the irregular particles are then positioned facing the fluid flow at three orientations which correspond to the particle’s minimum, median, and maximum area facing the flow resulting in the maximum, median, and minimum value of the drag coefficient, respectively. For each case, the drag coefficient is calculated through computational fluid dynamics by solving the continuity and Navier–Stokes equations for the fluid velocity field around the surface of the irregular particle and its multi-sphere representations. Figure 1 presents an example of representing the compact particle with various numbers of spherical elements at the orientation resulting in the maximum value of the drag coefficient for the actual shape. The multi-sphere representation of all the other particles at the three orientations is presented in the supplementary information document.

**Fig. 2:**
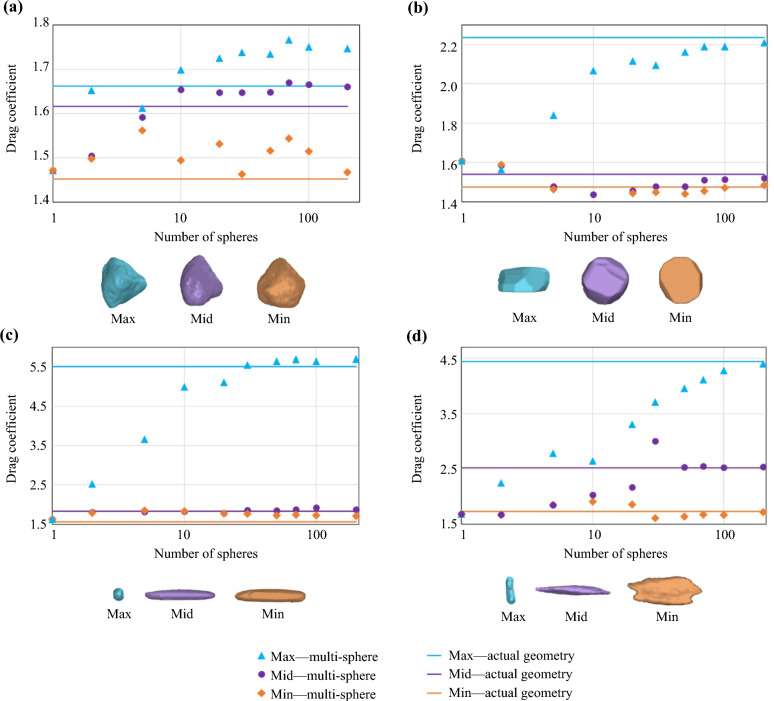
Comparison of the drag coefficient values for the actual shape and the multi-sphere representations for the particle with **a** compact, **b **flat, **c **elongated, and **d** bladed irregular shape positioned at orientations which result in the minimum, median, and maximum values of the drag coefficient for the actual shape

The drag coefficient of these multi-sphere clumps comprised of different numbers of spherical elements are compared to the drag coefficient of the actual shape at each of the three orientations of the minimum, median, and maximum area facing the flow. A summary of the results of the drag coefficient versus the number of spherical elements composing the multi-spheres is presented in Fig. 2.

For all cases, as the number of the spherical elements representing the irregular shape increases, the area facing the flow and the drag force also increases towards values closer to the values for the actual shape. Figure 2 shows that in most cases increasing the number of spherical elements of the clump representations results in a drag coefficient value closer to that of the actual shape. It is worth noting that representing the particle shape with its multi-sphere representation may create an artificial roughness on the surface of the particle which can influence the drag coefficient values.

For the three particle geometries of compact (Fig. 2a), flat (Fig. 2b), and elongated (Fig. 2c), the maximum difference between the values of the drag coefficient for the actual geometry and the multi-sphere representation using ten spherical elements is less than 18%. This can be considered acceptable for the purpose of the current study. So, particle shapes are represented as clumps of ten spherical elements in the CFD-DEM model to offer a compromise between the accuracy and the cost of the numerical modelling. The bladed particle geometry is not utilised in the current CFD-DEM model.

### Numerical model set-up

**Fig. 3:**
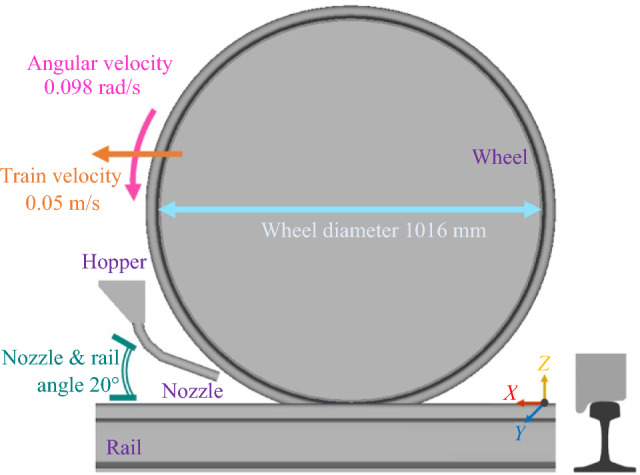
Geometry layout for the DEM numerical model along with a frontal view of the 60E1 rail and P8 wheel profiles

In DEM, the model is established based on the geometry of the full-scale wheel–rail rig at the Leonardo Centre for Tribology—The University of Sheffield [5]. The set-up consists of a wheel with a P8 profile, a diameter of ~ 1016 mm, and an angular velocity of 0.098 Rad/s as well as a rail with a 60E1 profile, a width of ~ 70 mm, a length of roughly three wheel diameters, and a translational velocity of 0.05 m/s. The sander nozzle is cylindrical with a 150 mm length and a 25 mm diameter which is placed at a 20° angle relative to the rail [5, 11]. All the solid components are modelled as perfectly rigid. A ~ 0.3 kg of sand particles with a Young’s modulus of 70 GPa, and a Poisson’s ratio of 0.25 is loaded inside the hopper [2]. The coefficient of restitution (CoR) of the sand particles which is defined in the DEM model to damp the sand particles’ bouncing of the wall is set to 0.5 [2, 25]. Figure 3 shows the details of geometry layout and dynamics of the DEM model.

The DEM modelling software EDEM (version 7.1.0) by Altair is used to perform the DEM simulations, with the time step set to 20% of the Rayleigh time step, which is calculated based on the size of the smallest particle. The application programming interface (API) in EDEM is used to implement the drag model into the DEM simulation. A total of 21 DEM simulations is reported here.

**Fig. 4:**
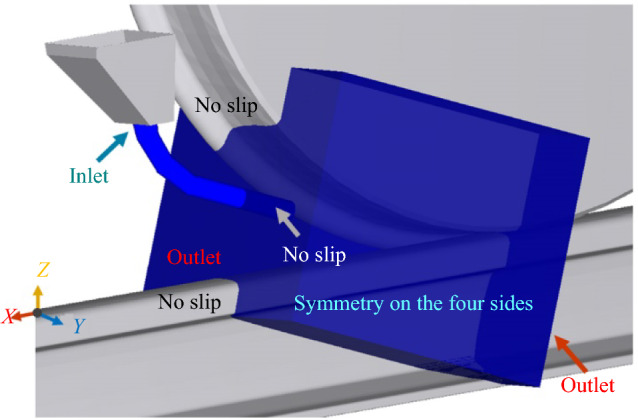
Geometry layout and boundary conditions for the CFD model

For the CFD model, the geometry is built according to the DEM model set-up, which comprises the sander pipes and nozzle connected to a rectangular cuboid. The boundary conditions are specified as known flow rate of 0.0034 kg/s at sander inlet, known ambient pressure at the two outlets, symmetry at the four sides of the rectangular cuboid, and no-slip (zero fluid velocity relative to the surface of the boundary due to fluid viscosity) on all the solid surfaces. The parts of the train wheel and rail which are located inside this cuboid are cut from the rectangular cuboid and act as moving walls with a rotational and a translational velocity of 0.098 rad/s and 0.05 m/s, respectively. Figure 4 shows details of the geometry layout and boundary conditions used for the CFD simulations.

The CFD simulation is carried out using ANSYS FLUENT (version 18.1) with the time step of 10^–6^ s and the convergence criteria set to 10^–6^. The governing equations of the fluid motion are discretised using the finite volume method (FVM) which are then solved using the semi-implicit method for pressure-linked equations (SIMPLE).

Mesh dependency analysis is carried out to make certain of the independence of the simulation results from the resolution of the computational grid. This will ensure a desirable numerical accuracy, but minimises the cost and time associated with the computational simulations. In conclusion, a mesh resolution of a little more than 14,000,000 grid elements with quadratic shapes are used here. A total number of four CFD simulations for mesh dependency analysis have been performed.

**Fig. 5:**
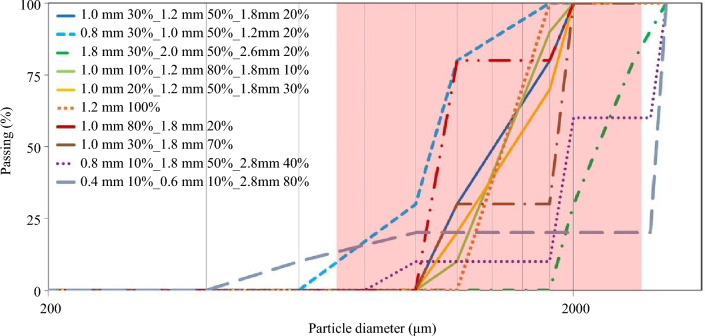
Particle size distribution graph used for the particle size investigation; the red area shows the size range currently accepted by GMRT 2461[15]

The characteristics of the sand particles including density, size distribution, and shape are the parameters studied here. Figure 5 shows the particle size distribution graph used for the particle size investigation. Table 1 presents the values investigated for these three parameters. The five particle candidates (including the benchmark) used for the particle density study include the British rail-sand with a density of 2600 kg/m^3^ as the benchmark along with recycled crushed glass, dolerite, coated alumina, and non-coated alumina with densities of 2500, 2900, 3700, and 3900 kg/m^3^, respectively.

**Table 1:** Parameters investigated for the parametric study

Parametric study	Particle Density (kg/m^3^)	Particle diameter (mm) (size distribution)	Coefficient of uniformity	Particle shape
Benchmark	2600	1.0 (30%), 1.2 (50%), 1.8 (20%)	1.5	Spherical
Particle density study	2500	1.0 (30%), 1.2 (50%), 1.8 (20%)	1.5	Spherical
	2900	1.0 (30%), 1.2 (50%), 1.8 (20%)	1.5	Spherical
	3700	1.0 (30%), 1.2 (50%), 1.8 (20%)	1.5	Spherical
	3900	1.0 (30%), 1.2 (50%), 1.8 (20%)	1.5	Spherical
Particle radius and size distribution study	2600	0.8 (30%), 1.0 (50%), 1.2 (20%)	1.6	Spherical
	2600	1.8 (30%), 2.0 (50%), 2.6 (20%)	1.3	Spherical
	2600	1.0 (10%), 1.2 (80%), 1.8 (10%)	1.3	Spherical
	2600	1.0 (20%), 1.2 (50%), 1.8 (30%)	1.5	Spherical
	2600	1.2 (100%)	1.0	Spherical
	2600	1.0 (80%), 1.8 (20%)	1.2	Spherical
	2600	1.0 (30%), 1.8 (70%)	1.8	Spherical
	2600	0.8 (10%), 1.8 (50%), 2.8 (40%)	2.0	Spherical
	2600	0.4 (10%), 0.6 (10%), 2.8 (80%)	5.0	Spherical
Particle shape study	2600	1.2 (100%)	1.0	Compact 10 elements
	2600	1.2 (100%)	1.0	Flat 10 elements
	2600	1.2 (100%)	1.0	Elongated 10 elements
	2600	1.2 (100%)	1.0	Truncated 8 elements
	2600	1.2 (100%)	1.0	Truncated 6 elements
	2600	1.2 (100%)	1.0	Truncated 4 elements
	2600	1.2 (100%)	1.0	Truncated 2 elements

### Results and discussions

**Fig. 6:**
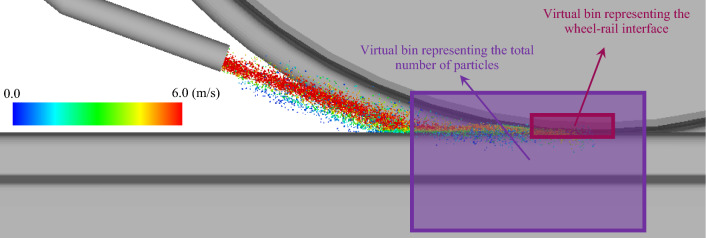
A snapshot of the numerical simulation of the rail-sanding showing GB rail-sand particle trajectories at a set timestep coloured by their velocities as well as the two virtual rectangular bins used to estimate the entrainment efficiency

The entrainment efficiency reported in the results obtained through numerical modelling is calculated as the ratio of the number of particles reaching the wheel–rail interface and landing inside a virtual rectangular bin to the total number of particles which have landed on the railhead inside another larger virtual rectangular bin. In our previous study, it was shown that the entrainment efficiency of the sand particles is dependent on the size and location of these rectangular bins but for the same size bins at the same locations, the trends of entrainment efficiency are comparable [11]. Figure 6 presents a snapshot of the numerical simulation showing particle trajectories coloured by their velocities as well as the two virtual rectangular bins used to estimate the entrainment efficiency. For the experimental investigations, the entrainment efficiency is measured by dividing the mass of the sand remaining on the rail’s surface after the wheel passed by the total mass of the sand used in the experiment [5]. It is worth mentioning that the numerical model simulates rail-sanding during the experiments performed in the laboratory [5] rather than during the actual train transport. Thus, the observations have some limitations, such as particles not being able to go through the wheel–rail interface due to the limited duration of the experiment, and the lack of longitudinal airflow.

### Effects of particle density on the entrainment efficiency

**Fig. 7:**
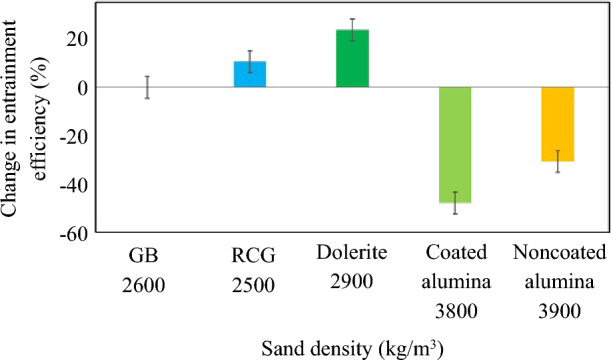
Percentage change in entrainment efficiency of the rail-sanding for four values of particle density compared to the benchmark case of 2600 kg/m^3^ density for GB rail-sand

Figure 7 shows the percentage change in entrainment efficiency of the rail-sanding for four sand density values compared to the benchmark case with a density of 2600 kg/m^3^ for GB rail-sand. It can be seen that as the sand particle density affects the entrainment efficiency of the rail-sanding with a ~ 50% decreases when the density increases by ~ 1000 kg/m^3^, while a constant air flow rate of 0.0034 kg/s is applied. The decrease in the entrainment efficiency due to using particles with higher densities can be remedied by increasing the air flow rate as previously reported [14]. The significance of this conclusion is that if other particle candidates [26] with higher or lower densities compared to the GB rail-sand are utilised for rail-sanding, the entrainment efficiency of the particles can be recovered by tuning the air flow rate. This can justify using other alternatives instead of Great Britain rail-sand due to the unsustainability of using sand or more favourable characteristics of other candidates [27].

### Effects of particle size, size distribution, and coefficient of uniformity on the entrainment efficiency

**Fig. 8:**
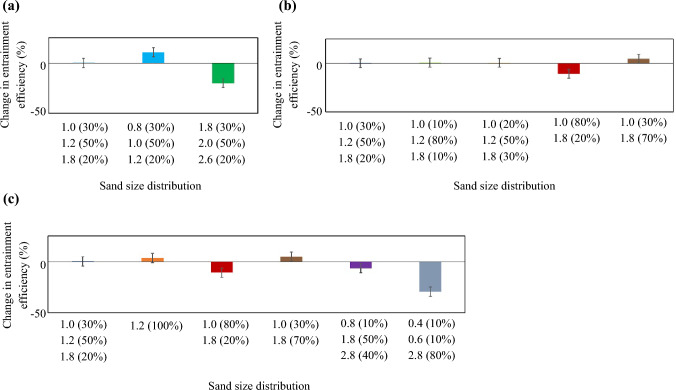
Percentage change in entrainment efficiency of the rail-sanding showing the effects of **a** particle size with constant size distribution, **b** particle size distribution, and **c** coefficient of uniformity of particles compared to the benchmark case with a size distribution of 30% of 0.5 mm radius, 50% of 0.6 mm radius and 20% of 0.9-mm radius

The percentage change in entrainment efficiency of the rail-sanding compared to the benchmark case with a size distribution of 30% of 0.5 mm radius, 50% of 0.6 mm radius and 20% of 0.9 mm radius is shown in Fig. 8. Here, three distinct effects can be studied: the effect of particles’ size shown in Fig. 8a, the effect of particles’ size distribution presented in Fig. 8b, and effect of particles’ coefficient of uniformity introduced in Fig. 8c. CoU is described as the ratio of the sieve size through which 60% by weight of the material passes to the sieve size that allows 10% by weight of the material to pass and the values are presented in Table 1.

Figure 8a shows that when the size distribution (the percentage representing the ratio of the coarse, the medium, and the fine sizes to the total amount of particles) remains the same, increasing the particle size lowers the entrainment efficiencies by ~ 20%. This results in the introduction of maximum particle size suitable for rail-sanding. The effect of particle size distribution for particles of the similar size is negligible on the entrainment efficiency as can be seen in Fig. 8b. Figure 8c illustrates that if CoU is higher than 2.5, the entrainment efficiency may decrease by ~ 20% which is in line with the guidelines presented in [15] stating “The uniformity coefficient shall be less than 1.5”.

### Effects of particle shape on the entrainment efficiency

**Fig. 9:**
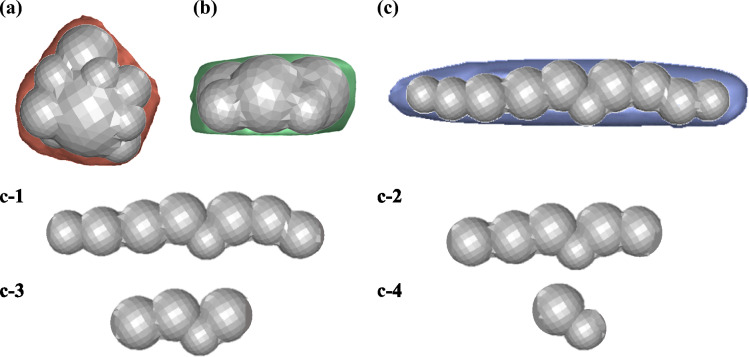
Shapes and multi-sphere representations of **a** compact, **b** flat, and **c** elongated particles; represented with ten spherical elements; and the elongated particle truncated by two, four, six, and eight, spherical elements (c-1 to c-4) symmetrically from both ends

Figure 9 shows the three particle shapes of compact (Fig. 9a**)**, flat (Fig. 9b**)**, and elongated (Fig. 9c**)** represented as multi-spheres of ten spherical elements in the DEM simulation. The elongated particle is then truncated by two, four, six, and eight spherical elements symmetrically from both ends to form particles presented in Fig. 9 c-1 to c-4**.** Particles with inherent elongated shapes can be produced such as the many RCG particles presented in [26, 27]. Bladed particles are not considered when studying the effect of shape as they are rarely encountered in natural sand and alumina particles [26].

**Fig. 10:**
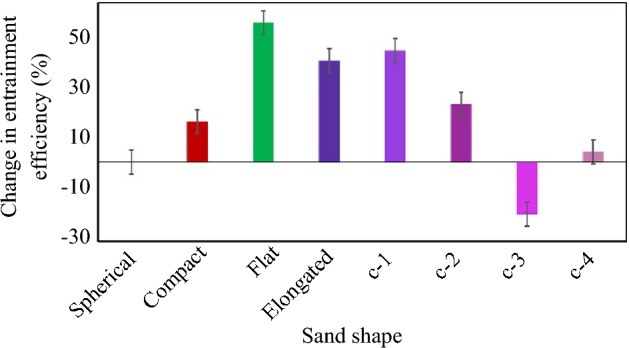
Percentage change in entrainment efficiency of the rail-sanding for three particle shapes compared to the benchmark case of spherical sand shape

The percentage change in entrainment efficiency of the rail-sanding process for the three sand shapes of compact, flat, and elongated as well as the truncated forms of the elongated particle by two, four, six, and eight spherical elements compared to the benchmark case with spherical sand is presented in Fig. 10. It can be concluded that among the three shape categories of compact, flat, and elongated particles of the same size, the flat particle results in the maximum entrainment efficiency with more than ~ 50% increase compared to the benchmark case. This is followed by the elongated and compact particles with more than ~ 30% and ~ 15% increase in the entrainment efficiency, respectively.

Comparing the elongated particle to its truncated forms, it can be seen that the effect of shortening the particles by two elements on the entrainment efficiency can be considered negligible, while the further truncation of the particle shape reduces the entrainment efficiency. One interesting observation is the considerable decrease in the entrainment efficiency for the case c-3 which can be explained by the overall shape of the truncated forms of the elongated particles. In Fig. 9, it can be seen that the multi-spheres named c-1, c-2, and c-4 appear to be relatively straight while the multi-sphere named c-3 is slightly curved into as S-shaped structure. The interaction of these S-shaped particles with the air medium around them is different compared to the straight particles resulting in the curved S-shaped particles to deviate more from the relatively linear trajectories usually followed by straight particles.

### A blind prediction with full-scale experiments

**Fig. 11:**
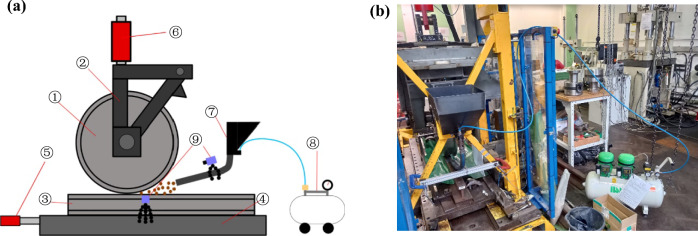
**a** Schematic of full-scale rig apparatus as used for this study; **b **actual photo of the full-scale rig

The simulations are blindly performed with limited information on the operational conditions of the full-scale experiments. The optimum case of 20° angle between the nozzle and the rail from [11] is used as the benchmark. Full-scale experiments are undertaken on a full-scale rig (FSR), a schematic of which is presented in Fig. 11a. The FSR is the same rig as used by Lewis et al. [5] for their study and utilises a very similar sanding apparatus, with the same compressor, hopper, valve, and hose and only having minor differences elsewhere (i.e. cameras used). Figure 11b shows the actual photograph of the full-scale rig.

The FSR is made of a train wheel ①, supported by a frame ②. The wheel is on top of a ~ 1 m piece of rail ③, which rests upon a bed ④ with low friction Polytetrafluoroethylene (PTFE) strips to allow for smooth longitudinal motion of the rail; the motion of the bed and rail is controlled by an actuator ⑤. A realistic wheel load can be applied with another actuator ⑥. The sanding apparatus is made up of a hopper and hose ⑦, with a stream of compressed air (supplied by an 8 bar, Bambi MD 150/500 compressor ⑧) picking up particles in the hopper and depositing them from the hose onto the rail. The two cameras ⑨ are installed to record the process.

The position of the hose is kept the same throughout (10º to the rail, 300 mm from the contact patch, and 20 mm above the rail) and the compressor pressure is adjusted for each material to deliver 2 kg/min. In order to enable particle entrainment for the alumina particles, a higher sanding rate is applied due to their higher densities. In all case studies, the rail moves in the longitudinal direction with a velocity of 0.1 m/s. The sanding system is activated during one wheel pass over 300 mm of rail, and crushed material is gathered from 150 mm of railhead after each test. In these experiments, and the entrainment efficiency of the rail-sanding process is defined as the ratio of the average mass of crushed sand remaining on 150 mm of the railhead after the wheel has passed to the ideal mass of sand which should theoretically remain on 150 mm of the railhead. Applying sand with a flow rate of 2 kg/min from the sander on 150 mm of the rail moving at 0.1 m/s results in the ideal mass of 50 g sand which should remain on the railhead to produce a 100% entrainment efficiency. Table 2 presents the values investigated for the full-scale experiments. It is worth mentioning that the inconsistencies in Table 2 are mostly due to the amount of material which is available.

**Table 2:** Values investigated for the full-scale experiments

Sand type	Starting pressure (bar)	Time (s)	Output (kg/min)	Average amount of sand remaining on rail (g)
British rail-sand	7	15	2.0	4.801
Recycled crushed glass 2 mm	7.5	15	1.9	1.627
Recycled crushed glass 1.18 mm	8	10	1.9	3.777
Recycled crushed glass 0.6 mm	8	5	2.0	2.116
Coated alumina	8	15	2.2	2.157
Non-coated alumina	7.5	15	2.0	0.757
Dolerite	7.5	3	2.0	4.484

**Fig. 12:**
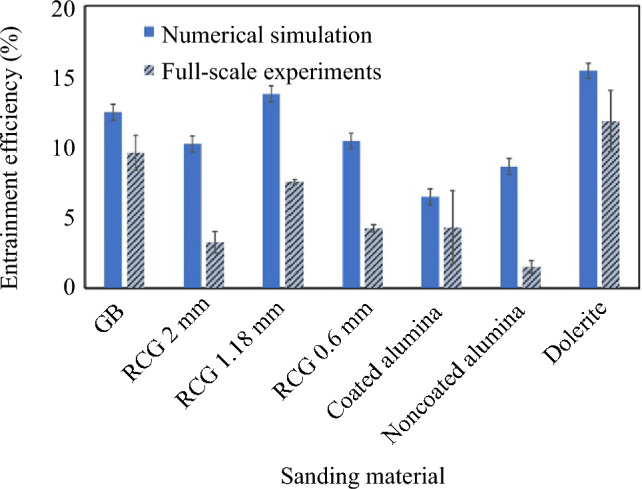
Entrainment efficiency of the rail-sanding for GB rail-sand and four rail-sand alternatives compared to the data obtained from full-scale test rig experiments

The entrainment efficiency of the rail-sanding for GB rail-sand and four rail-sand alternatives, namely recycled crushed glass (RCG) of three different sizes of 2 mm, 1.18 mm, and 0.6 mm [27], coated alumina, non-coated alumina, and dolerite [26] obtained through numerical simulations is blindly compared to the data obtained from full-scale experiments, and the values and trends are shown in Fig. 12. As previously explained, to calculate the entrainment efficiency of the sanding experiments, the average amount or sand remaining on the rail in grams (from Table 2) is divided by the ideal amount of 50 g.

It can be seen that the numerical simulation is able to accurately predict the trends in the entrainment efficiency with a maximum of ≤ 5% difference in the actual values which corresponds to a few milligrams of the material. The full-scale experiments resulting in lower values of particle entrainment efficiency compared to the numerical simulation is expected as even controlled environments can introduce undesirable effects to the experimental data compared to the ideal setting of numerical simulations.

Comparing the entrainment efficiency of the three sizes of RCG particles presented in Fig. 12, it can be seen that the maximum entrainment efficiency occurs for the 1.18 mm particles and increasing the particle size to 2 mm or decreasing it to 0.6 mm lowers the entrainment efficiency. This is in alignment with the guidelines presented in [15] indicating the optimum particle size distribution which is 0.71 mm ≤ particle diameter ≤ 2.8 mm.

In Fig. 12, a noticeable disparity between the values of entrainment efficiency obtained from numerical modelling using coated Alumina and non-coated Alumina particles can be seen. One reason for this disparity can be that in the numerical modelling the only difference between coated alumina and non-coated alumina particles is their corresponding densities, while in reality (as well as the experiments) coated alumina particle are treated with a coating that can affect their surface properties which in turn may change the entrainment efficiency of the experiments compared to the numerical model.

## Conclusions

In this research numerical simulations using CFD-DEM coupling were performed to model the rail-sanding process and present a parametric study on the sand particle characteristics by comparing particle entrainment efficiency obtained from the CFD-DEM model for various case studies to the benchmark case. It was shown that when the particle density increases by ~ 1000 kg/m^3^, the entrainment efficiency of rail-sanding is decreases by ~ 50% which can be rectified by increasing the air flow rate inside the sander. This can justify utilising other particle alternatives due to the unsustainability of using sand or more favourable characteristics of other candidates. The effects of particle size, size distribution, and coefficient of uniformity were shown to be as follows: increasing the particle size reduces the entrainment efficiencies by ~ 20%, particle size distribution (within the accepted range of the standard) has negligible effect on the entrainment efficiency, and increasing the coefficient of uniformity higher than 2.5 decreases the entrainment efficiency by a maximum of ~ 20%. Particle shape highly affects the entrainment efficiency with more than ~ 50%, ~ 30%, and ~ 15% increase for flat, elongated, and compact particles compared to the spherical shape, respectively. A blind comparison of the numerical simulation results to full-scale experiments for GB rail-sand and four rail-sand alternatives has also been performed. The numerical simulation was shown to be capable in accurately predicting the trends of the entrainment efficiency with only a ≤ 5% difference compared to the actual values obtained from full-scale tests.

## Footnotes

1 In the railway industry “adhesion” or “adhesion coefficient” is defined as the amount of traction present when the wheel–rail contact enters partial slip. In this paper, the terms are used interchangeably.
